# Comparison of oral and intravenous Alfacalcidol in chronic hemodialysis patients

**DOI:** 10.1186/1471-2369-15-27

**Published:** 2014-02-04

**Authors:** Myriam Lessard, Denis Ouimet, Martine Leblanc, Annie-Claire Nadeau-Fredette, Robert Bell, Jean-Philippe Lafrance, Vincent Pichette, Michel Vallée

**Affiliations:** 1 Hôpital Maisonneuve-Rosemont, 5415 Boulevard de l′Assomption, Montréal H1T 2 M4, Québec, Canada

**Keywords:** Alfacalcidol administration, End-stage renal failure, Hyperparathyroidism

## Abstract

**Background:**

Activated vitamin D is the mainstay of treatment for secondary hyperparathyroidism (SHPT) in chronic hemodialysis patients. However, the optimal route of administration is still debated. The aim of our study was to compare efficacy of oral *vs* intravenous (IV) administration of alfacalcidol in hemodialysis. A secondary objective was to determine the cost-effectiveness advantage of oral administration.

**Methods:**

Eighty-eight chronic hemodialysis patients receiving IV alfacalcidol three times a week were included in the study. All were switched to the same dose of alfacalcidol given orally three times a week during the hemodialysis session. A budget impact analysis was performed.

**Results:**

Mean patient age was 64 years old and 43% were males. The mean alfacalcidol dose administered was 2.1 μg three times a week. After three months, serum parathormone (PTH) levels decreased from 80 to 59 pmol/L (p = 0.001) and total serum calcium levels increased from 2.34 to 2.40 mmol/L (p = 0.002). After six months, total serum calcium levels were still significantly higher. Alfacalcidol dosage was significantly decreased during study period; the mean reduction was 0.44 μg per dose. Finally, oral administration was associated with an annual cost reduction of 197 678$CAN and an annual nursing time reduction of 25 days.

**Conclusion:**

Our findings support that switching IV to oral administration of alfacalcidol during hemodialysis sessions may lead to a similar control of SHPT with lower doses of activated vitamin D. This is a good strategy for optimizing compliance and may allow a dose reduction because of a greater efficacy to suppress PTH. Oral administration also has significant cost-effectiveness advantages.

## Background

Secondary hyperparathyroidism is an early and common complication of chronic kidney disease (CKD). This phenomenon is caused by several factors, such as calcitriol deficiency, progressive hyperphosphatemia, relative hypocalcemia and parathormone (PTH) skeletal resistance [1]. Prevalence of native vitamin D deficiency exceeds 80% in advanced CKD population [2]. We know that vitamin D plays a central role in phosphocalcic and bone metabolism in the general and the CKD population. However, 25-vitamine D needs to undergo 1-α-hydroxylation before being active. Because kidneys are the primary site for this hydroxylation, active 1,25-vitamin D or calcitriol is consequently deficient in CKD and also contribute to PTH elevation [2]. Down regulation of vitamin D receptor (VDR) and decreased calcium sensing receptor (CaSR) on parathyroid cells have also been implicated in secondary HPT pathogenesis [3,4]. Such changes alter bone remodelling, may exacerbate osteoporosis and even lead to osteitis fibrosa, a pattern of renal osteodystrophy. Chronic excessive PTH levels are frequent in end-stage renal disease (ESRD) and are associated with parathyroid hyperplasia and eventually autonomous secretion. The first intervention to tackle this problem is to keep serum phosphorus level close to the normal range with diet, good dialysis and phosphate binders (PB). Once serum phosphorus is controlled, administration of activated vitamin D is the mainstay of treatment of SHPT in chronic hemodialysis (HD) patients. There are several mechanisms by which vitamin D analogs regulate PTH. Vitamin D analogs increase serum calcium levels by enhancing intestinal calcium absorption resulting in a decrease in PTH levels [5]. These analogs also produce a direct inhibition of PTH gene transcription in parathyroid cells [5]. Finally, activated vitamin D could also move the set point for calcium in parathyroid glands to the left side, which means that PTH secretion is reduced in response to serum calcium levels. Activated vitamin D can be administered orally, daily or intermittently and intravenously (IV three times a week during HD sessions).

Studies comparing oral and IV routes of administration for active vitamin D have found conflicting results [6-13]. The optimal route of administration for activated vitamin D is still debated [13] because there are limited randomized placebo-controlled studies that directly compare pulse-oral to pulse-IV activated vitamin D administration for the treatment of SHPT in ESRD.

In our HD center, IV administration is mostly used for compliance concerns in patients with uncontrolled SHPT with daily oral regimen. Moreover, alfacalcidol is preferred to calcitriol because of a lower cost. Calcitriol is thus reserved for patients with hepatic dysfunction, hypoparathyroidism following parathyroidectomy or refractory SHPT. Most previous clinical studies comparing routes of administration of activated vitamin D in HD population were conducted with calcitriol and only a few studies considered alfacalcidol administration [11,14,15].

The primary objective of our study was to compare efficacy and side effects of oral versus IV in-center administration of alfacalcidol in HD. A secondary objective was to evaluate the cost-effectiveness of oral compared to IV regimen. Our hypothesis was that intermittent oral administration of alfacalcidol during HD sessions could be at least as effective as IV administration for SHPT control in regards to KDIGO guidelines [16]. Moreover, IV switch to oral administration could also represent a cost-effective strategy.

## Methods

### Cohort definition

We performed a retrospective observational study among subjects on chronic HD treatment for at least six months at Maisonneuve-Rosemont Hospital. All subjects treated with the same dose of alfacalcidol given IV three times a week for the last three months were included. We excluded patients with intestinal malabsorption or hepatic disease.

### Assessment of outcome

Oral alfacalcidol was started at the same dose as prior IV dose and administered by nursing staff at the end of each HD treatment. Alfacalcidol dose was then adjusted according to KDIGO recommendations [16].

Collected demographic data for each patient included: age, sex, ESRD etiology, HD vintage and history of parathyroidectomy. Monthly biochemical parameters were collected for three months before and up to six months after the switch from IV to oral thrice-weekly alfacalcidol. Biochemical and pharmacological data included: serum calcium, phosphorus, albumin, bicarbonates, magnesium, intact PTH, 25(OH)D_3_, 1.25(OH)_2_D_3_, alkaline phosphatase, phosphocalcic product, calcium concentration dialysis bath, dose of phosphate binders (calcium carbonate, sevelamer hydrochloride, lanthanum carbonate, aluminium/magnesium based binders), dose of cinacalcet as well as alfacalcidol.

### Pharmaco-economic analysis

Annual costs and nursing time were calculated for both oral and IV routes of administration. Ten patients were identified for the evaluation of the time and motion study required for drug administration. Annual costs included drug price, medical supplies and nursing time and were extrapolated for the six-month study and nursing time evaluation. Cost for nursing time were calculated by multiplying nursing time by average hourly wage for nephrology nurses in Quebec, including social benefits, obtained from the Quebec Ministry of Health [17].

### Statistical analysis

Parameters at baseline, 3 and 6 months after the switch were compared using a Student’s paired t-test.

### Ethical considerations

The Research and Ethics Committee of Maisonneuve-Rosemont Hospital approved the study.

## Results

### Patient characteristics

The cohort consisted of 88 patients with a mean age of 64 ± 14 years; 43% were males. They represented 24% of the entire HD cohort. The mean IV alfacalcidol dose was 2,10 ± 0.86 μg three times a week at study entry and before the switch to oral alfacalcidol. Baseline characteristics, including ESRD etiologies and initial serum biochemistry results are summarized in Table 1. No patients died or were lost to follow-up during the study.

**Table 1:** Characteristics of patients at baseline (N = 88 patients)

**Characteristics**	**Mean or percentage**
Age (year), mean (SD)	64 (14)
Sex (male), n (%)	38 (43.2)
IV dosage of alfacalcidol, mean (SD)	2.1 μg 3 times a week (0.86)
ESRD etiology, n (%)	
Diabetic nephropathy	30 (34)
Hypertensive nephropathy	30 (34)
Glomerular disease	15 (17)
Others	13 (15)
Cinacalcet use, n (%)	30 (34)
Phosphate binders use, n (%)	
Calcium based	64 (72)
Sevelamer	50 (57)
Lanthanum	9 (10)
No phosphate binder	8 (9)
25 vitamin D use, n (%)	0 (0)

Data are presented as number (percentage) or mean (standard deviation), SD: standard deviation.

### Effects on serum biochemistry and drug dosage adjustments

Three months after switching from IV to oral alfacalcidol, total serum calcium levels increased from 2.34 to 2.40 mmol/L (p = 0.002), whereas serum PTH levels significantly decreased from 80 to 59 pmol/L (p < 0.001) and serum phosphorus levels did not change significantly (Table 2). There was a small decrease of alfacalcidol dosage from baseline to three months (p = 0.04). However six months after the switch, the decrease of alfacalcidol dosage became statistically significant compared to baseline (p < 0.001). There had been a 21% reduction of dosage, which corresponded to a mean reduction of 0.54 μg per dose three times a week. At six months, the increase in total serum calcium was still significant (p < 0.001) but no episodes of hypercalcemia were reported. However, serum PTH and phosphorus levels were not significantly different from baseline values. Concomitantly, serum 25(OH)D_3_ levels was clinically unchanged, although statistically reduced (p < 0.001) while 1.25(OH)_2_D_3_ levels significantly increased from 32 at baseline to 40 pmol/L at six months (p = 0.007). Moreover, total alkaline phosphatase level significantly decreased three months after the switch; this reduction was still significant at six months when compared to baseline levels.

**Table 2:** Biochemical parameters and alfacalcidol dose at baseline, at three months and at six months after the switch to oral administration

**Serum parameters**	**Baseline IV alfacalcidol administration**	**3 months after switch to oral alfacalcidol**	**6 months after switch to oral alfacalcidol**
Calcium, mmol/L (SD)	2.34 (0.17)	2.40 (0.18)**	2.43 (0.17)**
Phosphorus, mmol/L (SD)	1.49 (0.44)	1.58 (0.44)	1.57 (0.50)
Ca x PO_4_ (SD)	3.49 (1.06)	3.78 (1.07)*	3.77 (1.18)
Intact PTH, pmol/L (SD)	79.7 (59)	58.5 (56)**	78.2 (77)
Alkaline phosphatise (SD)	142.3 (103)	127.7 (96)*	130.2 (103)*
25(OH)D_3_, nmol/L (SD)	55 (20)	57 (24)	50 (21)**
1,25(OH) _2_D_3_ (pmol/L) (SD)	32 (19)	38 (17)*	40 (18)*
Mean alfacalcidol dose (SD)	2.1 μg 3 times a week (0.86)	1.87 μg 3 times a week (0.92)*	1.56 μg 3 times a week (0.79)**

*Ca x PO*_*4*_, Phosphocalcic product, *SD*, Standard deviation.

*P < 0.05 compared to baseline.

**P < 0.001 compared to baseline.

There was no significant change in the cinacalcet dosage of the 30 patients under this medication during the study period. There was no significant change in phosphate binders dose; although six patients had a slight increase, eight patients had a small decrease in their phosphate binder dose and eight patients did not take phosphate binder during the study period. There was no change in dialysate calcium concentration during the study period that remains at 1.25 mmol/L for all patients.

### Pharmaco-economic analysis

As shown in Table 3, annual costs related to nursing time and medical supplies were much lower with oral alfacalcidol regimen. The annual cost reduction was 7270$CAN for the entire cohort or 82.61$CAN per patient. More importantly, there was a ten-fold decrease in annual drug cost per patient, from 2493$CAN for IV alfacalcidol to 329$CAN for oral alfacalcidol. Thus, in our unit, compared to IV, oral administration was associated with a total annual cost reduction of 197 678$CAN (2246$CAD per patient) and an annual nursing time reduction of 25 days.

**Table 3:** Estimated annual costs per patient according to alfacalcidol administration regimen

	**Cost per patient CAD$**	
	**IV administration**	**Oral administration**
Medical supplies	31.20	0
Nursing time	64.20	12.80
Drug	2492.80	329.20
Total	2588.20	342.00

## Discussion

The primary objective of our study was to demonstrate that oral intermittent administration of alfacalcidol in HD was at least as effective as IV administration in controlling PTH. Our results demonstrated that thrice-weekly oral alfacalcidol administration is more effective than an equivalent thrice-weekly IV regimen. We also found that oral administration of alfacalcidol was associated with a significant increase of serum calcium levels, a lower serum PTH and total alkaline phosphatase levels. Reduction of serum PTH and total alkaline phosphatase are usually predictive of better bone remodelling control in SHPT [18]. Such an apparent increased efficacy was associated with a significant decrease in alfacalcidol oral dose during the study period compared to prior IV dose. To our knowledge, this is the first study to show such findings. No episode of hypercalcemia was reported during the study period, although this remains a long-term possibility if alfacalcidol dosage is not carefully adjusted.

Several studies have shown that alfacalcidol is effective for SHPT control when compared to others forms of activated vitamin D [19-22]. Mitwalli et al. [14] reported in 2000 that oral intermittent administration of alfacalcidol was at least as effective as IV administration in controlling serum PTH. Although all patients were initiated on alfacalcidol 1 mcg three times per week, the final doses within the oral and IV groups were not reported by the authors. Some studies propose that pulse alfacalcidol oral therapy is as effective as daily oral administration in regards to PTH suppressive effect [11,15].

Some studies suggest that intermittent administration is associated with a lower incidence of hypercalcemia and may suppress PTH with more efficacy than daily administration of activated vitamin D [23,24]. One study showed that IV intermittent calcitriol therapy is the best route of administration in regards to PTH suppressive effect [6]. Another study also suggested that IV calcitriol administration in the early stage of kidney failure can prevent the exacerbation of parathyroid hyperplasia and delay the apparition of nodular hyperplasia [4]. Others demonstrated that IV calcitriol treatment has a superior effect on bone remodelling [7,8]. However, more recent publications have shown that high-dose intermittent oral administration may be as effective as IV administration in treating SHPT [1,9-11]. Zhou et al. published in 2009 a meta-analysis of randomized controlled trials comparing intermittent IV and oral calcitriol in chronic HD patients [12]. Six studies were considered for their meta-analysis and they showed that there were no significant differences between the two routes of administration in regards to efficacy and adverse effects (hypercalcemia and/or hyperphosphatemia).

The response to activated vitamin D therapy may also be related to SHPT severity instead of the route of administration of calcitriol [9,25-27]. Obviously, activated vitamin D efficacy is also linked to patient’s compliance with the drug regimen [28]. Nonetheless, the optimal route of administration of activated vitamin D is still debated [13] because there are limited randomized placebo-controlled studies that directly compare pulse-oral to pulse-IV activated vitamin D administration for the treatment of SHPT in ESRD.

Although it has not been demonstrated, we could postulate that oral alfacalcidol higher efficacy is partially related to the “first hepatic pass effect”. For example, we can compare this phenomenon to Amiodarone class III antiarrhythmic action which is mostly related to the pharmacologic action of the hepatic metabolite [29,30]. Similarly, Alfacalcidol is a pro-drug that must undergo hepatic 25-hydroxylation before being active [5]. This hydroxylation may be greater when alfacalcidol is administered orally avoiding rapid peripheral catabolism. This phenomenon could lead to higher levels of 1.25(OH)_2_D_3_, higher intestinal calcium absorption, slightly higher levels of serum calcium and better control of SHPT. However, efficacy could be different for a vitamin D analog that would not be a pro-drug with hepatic metabolism. For sure, we cannot exclude some tubing adsorption with IV administration. Another potential explanation comes from the fact that IV administration of alfacalcidol yields a peak serum of medication that is faster and higher than the oral administration of alfacalcidol. Thus, it is possible that this phenomenon increases the catabolism of alfacalcidol and 1.25(OH)_2_D_3_.

Our secondary objective was to study the cost-effectiveness of oral compared to IV alfacalcidol administration in HD. Oral administration is significantly cheaper, which is consistent with literature [31]. It is well known that SHPT in CKD patients has a significant economic burden and must soon be recognized and treated [31]. Savings are mainly related to lower drug price and reduced nursing time, which is a major issue in a context of nursing shortage.

Since our study is not a randomized control trial, we cannot exclude that the observed results are due to unmeasured or unknown factors, other than the route of alfacalcidol administration.

## Conclusions

Although some studies have suggested that intermittent IV administration of activated vitamin D analogs may be associated with a better control of SHPT, it is not the case for alfacalcidol, a pro-drug that needs hepatic hydroxylation before being active. Moreover, our findings suggest that intermittent oral administration is even more effective than equivalent IV dosage in regards to PTH suppressive effect. For maintaining serum PTH levels within target limits or for compliance purposes, intermittent oral administration of alfacalcidol in an HD unit is a much more cost-effective strategy. In our unit, for 88 patients for whom intermittent activated vitamin D administration was considered medically appropriate, such a strategy represents an annual saving of 197 678$CAN and significantly reduces nursing time required for drug administration during HD sessions.

## Competing interests

The authors declare that they have no competing interests.

## Authors’ contribution

MV, ML and RB conceived of the study and participated in its design and coordination and helped to draft the manuscript. JPL participated in the design of the study and performed the statistical analysis. DO, ML, ACNF and VP participated in the design of the study and coordination and helped to draft the manuscript. All authors read and approved the final manuscript.

## Pre-publication history

The pre-publication history for this paper can be accessed here:

http://www.biomedcentral.com/1471-2369/15/27/prepub
